# Analysis of gene expression patterns by microarray hybridization in blood mononuclear cells of SLA-DRB1 defined Canadian Yorkshire pigs

**DOI:** 10.1186/1756-0500-1-31

**Published:** 2008-06-23

**Authors:** Maria I Nino-Soto, Razi Jafari Jozani, Byram Bridle, Bonnie A Mallard

**Affiliations:** 1 Department of Pathobiology, Ontario Veterinary College, University of Guelph, Guelph, Ont., N1G 2W1, Canada; 2 Faculty of Veterinary Medicine, University of Tehran, Tehran, Iran

## Abstract

**Background:**

The Swine Leukocyte Antigen (SLA) system encodes molecules for self-nonself discrimination and is associated with immune responses and disease resistance. Three lines of pigs defined by their SLA-DRB1 alleles were developed at the University of Guelph for xenotransplantation and immune response studies. The aim of this project was to explore the potential association between defined SLA-DRB1 alleles and gene transcriptional patterns of other immune-related genes in blood mononuclear cells.

**Findings:**

Three SLA-DRB1 alleles were characterized using a RT-PCR-based sequencing method. The loci represented included a new allele, DRB1*04ns01. Next, microarray heterologous (bovine-porcine) hybridization together with qPCR were used to explore differential gene expression between SLA-DRB1-defined groups. Microarray analysis showed significant (p < 0.01) differential expression for 5 genes, mostly related to inflammation. Genes varied according to the comparison analyzed. Further testing with qPCR revealed the same trend of differential expression for 4 of the genes, although statistical significance was reached for only one.

**Conclusion:**

A new SLA-DRB1 allele was characterized. A potential association was found between SLA-DRB1 alleles and inflammation-related genes. However, the influence of other genes cannot be ruled out. These preliminary findings agree with other studies linking MHC haplotypes and inflammation processes, including autoimmune disease. The study provides an initial view of the biological interactions between the SLA complex and other immune-related genes. Future studies will focus on characterization of SLA-haplotypes associated with these particular alleles and the dynamics of the immune response to antigenic challenges.

## Findings

The highly polymorphic MHC-encoded molecules are crucial for self-nonself discrimination in vertebrates. They constitute the major barrier for transplantation, contain numerous genes involved in immunological and non-immunological functions and are associated with resistance or susceptibility to various diseases. The two main classes, I and II, are involved in antigen presentation to T-cells. However, a large number of the genes in the MHC, like class III genes, are not directly related to this function [1,2]. A total of 152 loci have been annotated within this region. In pigs, known as the SLA, the DRB genes show extensive polymorphism in exon 2 and the 135 available sequences identified to date are distributed into at least 10 confirmed allele groups [3].

Different SLA haplotypes have been associated with variation in immune response and disease, as well as reproduction and production traits [4]. Therefore, SLA-defined pigs constitute an invaluable resource to study immune response, disease resistance and production traits, as well as an important large animal model for biomedical research [5,6]. Three lines of commercial Yorkshire pigs with defined SLA-DRB1 genotypes were produced at the University of Guelph for xenotransplantation and immune response research [7,8]. The aims of this study were to characterize the SLA-DRB1 alleles in these three pig lines and explore differential transcriptional activity between the three groups using heterologous (bovine probes – porcine targets) cDNA microarray and qPCR.

### Animals and samples

Animal use was approved by the Animal Care Committee of the University of Guelph. Thirty-five pigs were included in the study (n = 6 for microarray analysis and n = 29 for qPCR). Pigs came from crossings of an outbred population selected for only by specific SLA-DRB1 alleles. Age of pigs ranged between 3–6 months and all pigs were in good general health at the time of sampling. Venous blood was collected in EDTA coated BD Vacutainer^® ^collection tubes (BD – Canada, Oakville, ON, Canada) and processed immediately after collection. MNCs were isolated using Histopaque-1077 (Sigma-Aldrich Canada Ltd., Oakville, ON, Canada) and total RNA was extracted using TRIzol™ reagent (Invitrogen Canada Inc., Burlington, ON, Canada). Total RNA was treated with DNA-*free *(Ambion Inc., TX, USA) to eliminate genomic DNA contamination. Concentration and quality were assessed with an Agilent 2100 Bioanalyzer (Agilent Technologies Inc., Santa Clara, CA, USA).

### SLA-DRB1 alleles characterization

**Figure 1:**
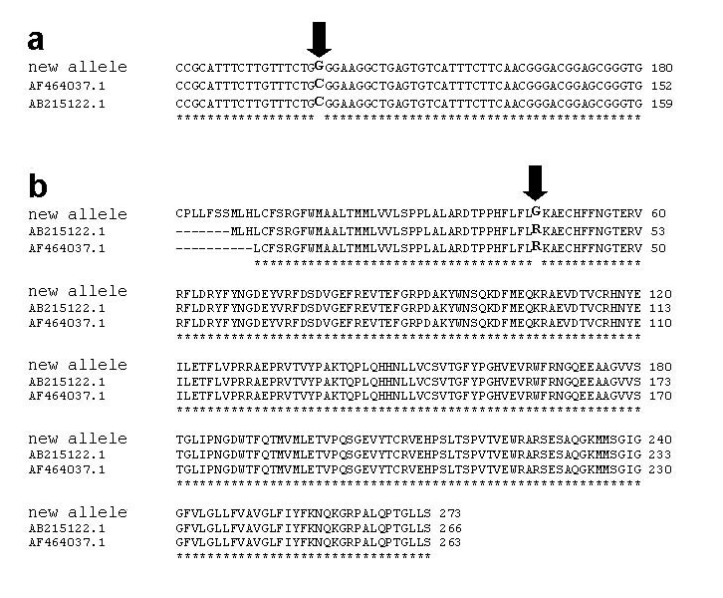
**Nucleotide and protein sequence alignment of SLA-DRB1 alleles**. Multiple sequence alignment for (a) nucleotide and (b) protein of published SLA-DRB1*0403 alleles (designated by their GenBank accession numbers) and *04ns01 (new allele). The black arrows mark (a) the point mutation and (b) the corresponding amino acid residue substitution.

The SLA-DRB1 alleles were characterized according to Ho et al. (2006). Briefly, total RNA was reverse transcribed with the ThermoScript™ RT-PCR system using oligo d(T) primers (Invitrogen). A specific SLA-DRB1 coding region was amplified with PfuUltra™ Hotstart High-Fidelity DNA Polymerase (Stratagene, La Jolla, CA, USA) using a final concentration of 3 mM of MgCl_2 _and annealing temperature at 55°C. PCR products were gel-extracted with the QIAquick Gel Extraction Kit (Qiagen, Mississauga, ON, Canada) and purified PCR products were cloned using the Zero Blunt^® ^TOPO^® ^PCR cloning kit (Invitrogen). At least 7 colonies per animals were sent for sequencing. Each allele was characterized by sequencing using both forward and reverse primers, from at least two pigs per litter. The complete coding sequence was obtained by overlapping the forward and reverse fragments. Comparison with currently available sequences (GenBank and EBI-IPD-MHC, SLA section databases) was performed using BLAST [9] and ClustalW [10]. Two lines carried the SLA-DRB1*0502 and SLA-DRB1*0701 alleles respectively (Smith et al, 2005) as determined by a 100% homology between the sequences obtained from test samples and the corresponding published sequences. The third line had a novel allele, differing in one bp at position +118 in exon 2 with SLA-DRB1*0403. This difference corresponds to a point mutation substituting a cytosine for a guanine, which translates in the substitution of an arginine for a glycine residue in the protein encoded by this new allele (Figure 1). Sequences were submitted to GenBank [Genbank: EU087426, EU087427 and EU087428] and the EBI IPD-MHC (SLA section) which resulted in the assignment of the SLA-DRB1*04ns01 provisional name to the new allele, approved by the MHC Nomenclature Committee.

### cDNA Microarray experiments

**Figure 2:**
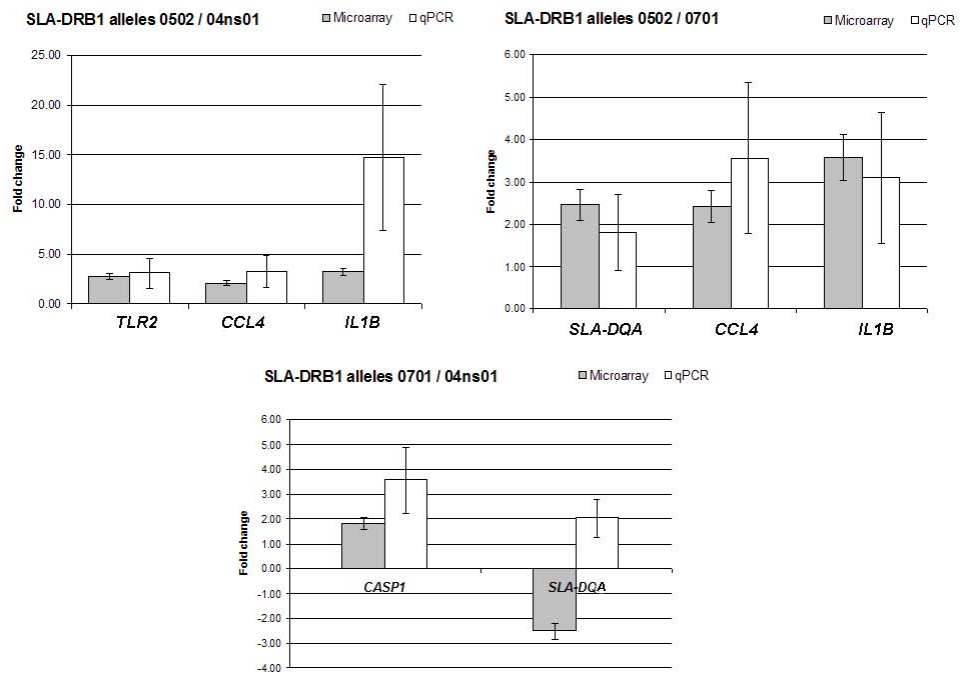
**Differential transcriptional activity detected by cDNA microarray hybridization and qPCR**. Mean fold changes in transcript quantification obtained by cDNA microarray hybridization () and qPCR (□). a) Microarray (n = 2 per group) and qPCR (*0502, n = 9; *04ns01, n = 14) results for the comparison between SLA-DRB1*0502 and *04ns01. b) Microarray (n = 2 per group) and qPCR (*0502, n = 9; *0701, n = 6) results for the comparison between SLA-DRB1*0502 and *0701 alleles. c) Microarray (n = 2 per group) and qPCR (*0701, n = 6; *04ns01, n = 14) results for the comparison between SLA-DRB1*0701 and *04ns01.

Complete and detailed information on microarray experimental protocols, the datasets and the platform were submitted to GEO (accession number GSE7908). Experiments are described according to the MIAME standard [11]. Heterologous hybridizations (bovine probes – porcine targets) were performed to compare the three groups representing defined SLA-DRB1 alleles (n = 2 pigs per group). A loop design was used for reciprocal comparisons. Six comparisons with dye-swap on the same slide were performed for a total of 12 microarrays. Data was analyzed using Acuity 4.0 (Molecular Devices Corp., Sunnyvale, CA, USA) and normalized with the LOWESS algorithm [12]. After normalization, data was filtered based on flags, percentage of saturated pixels, background and intensity uniformity, and signal to noise ratio. The log-ratios of expression were calculated as the base 2 logarithm of the ratios of background-corrected intensity medians of red dye over green dye intensities. A gene was considered to be differentially expressed if it had an absolute value of log-intensity ratio higher or equal to 0.8, representing a fold-change of 1.7 in transcript quantity. Statistical analysis was performed using the Student's t-test with FDR correction for multiple comparisons [13]. Statistical significance was set at p = 0.01. We had previously validated the use of this in-house immune-endocrine bovine microarray with porcine targets [14]. In this study, hybridization resulted in ~90% positive signals (~170 features) in agreement with those previous observations. However, the presence of positive signals of hybridization does not imply that all spots will provide valid results. As previously mentioned, we performed careful filtering to ensure that only consistent data was subject to further analysis. Results from microarray data analysis are summarized in Table 1 and Figure 2. The *0502 allele group showed higher transcriptional activity for *CCL4 *and *IL1B *in all comparisons. The *0701 allele group showed less *SLA-DQA *transcripts in all comparisons. Transcripts amounts for *TLR2 *and *CASP1 *were higher in the *0502 and *0701 allele groups respectively, when compared to the *04ns01 group. The small number of genes consistently detected as differentially expressed reflects the tendency of heterologous hybridization to reduce the effective size of a given microarray. Although optimal results are obtained with homologous hybridizations, the use of heterologous microarray hybridization is still a valid approach to assess gene expression profiles given that measures are taken to preserve the quality of the data obtained [15]. In addition, results were verified using qPCR as stated in the next section.

**Table 1:** Summary of results from cDNA microarray and qPCR data analysis

	**MICROARRAY**			**qPCR**		
Gene	Fold-change ^a^	Ratio^b^	p-value ^c^	Fold-change ^a^	Ratio	p-value

**Comparison A: SLA-DRB1 alleles 0502/04ns01**						

*TLR2*	2.76	1.468	< 0.0001	3.10	1.65	0.0586
*CCL4*	2.10	1.075	0.0010	3.27	1.71	0.2880
*IL1B*	3.21	1.683	0.0025	14.72	3.88	0.0322
**Comparison B: SLA-DRB1 alleles 0502/0701**						

*SLA-DQA*	2.46	1.303	0.0038	1.8	0.85	0.3569
*CCL4*	2.41	1.271	0.0158	3.55	1.83	0.2926
*IL1B*	3.57	1.837	0.0079	3.1	1.64	0.4168
**Comparison C: SLA-DRB1 alleles 0701/04ns01**						

*CASP1*	1.79	0.840	0.0010	3.56	1.83	0.0651
*SLA-DQA*	-2.52	-1.335	0.0035	2.02	1.02	0.4667

^a ^Fold change = 2 ^(Log-ratio) ^or (-1) 2 ^(Log-ratio)^; ^b ^Median of the lowess-normalized log-ratio of intensity; ^c ^p-value with FDR correction for multiple comparisons.

Numerous associations have been established in swine between SLA haplotypes and features such as immune response and disease [16,17], reproduction [18] and production traits [19]. Many of these traits are not directly regulated by individual SLA genes but could rather be under the influence of non-classical MHC genes or controlled by downstream pathways yet to be described. The involvement of other closely linked genes, whose variants are in linkage disequilibrium (LD) can not be discarded [20,21]. For example, it has been found that differential expression of *LTB *(also known as TNF beta) in MHC class II-defined B cell lines is associated with certain MHC class II haplotypes but not others. This association could be explained by LD between *LTB *and MHC haplotypes or by the influence of polymorphism in the MHC class II molecules and their interactions on the control of gene expression [22]. Another example is represented by *BRD2 *in humans. This transcription factor, without an established immune function and located in the MHC class II region, is strongly linked to the MHC in most vertebrates [23].

Although it is not possible from the results in this study to establish a direct causal relationship between particular SLA-DRB1 alleles and differential transcription of inflammatory genes observed, it is undeniable that there seems to be an association. These observations will be better explained by the characterization of the haplotypes linked to these alleles and further exploration of the immune response in animals with defined MHC haplotypes.

### Quantitative RT-PCR

To verify differential expression observed in the microarray data, qPCR calibrator-normalized relative quantification with efficiency correction in the LightCycler^® ^1.5 system and the Relative Quantification Software v. 1.0 (Roche Diagnostics, Laval, QC, Canada) were used. *RPL19 *was tested for variability among samples and selected as reference gene. Specific PCR conditions and primers are described in Table 2. Total RNA samples (*0502, n = 9; *0701, n = 6 and *04ns01, n = 14) were reverse transcribed using SuperScript III (Invitrogen). The qPCR was performed using LightCycler^® ^FastStart DNA Master SYBR Green I (Roche). Relative standard curves for target and reference genes were created using dilution series with six 10-fold dilutions in triplicates. One of the dilutions was used as calibrator. Replicate determinations were performed using independent reverse transcription reactions. Results are reported as normalized ratio of target/reference concentrations. Data sets from qPCR were analyzed with a general linear model [24] using the SAS system for Windows v 9 (SAS Institute Inc., Cary NC, USA). A log transformation was used to normalize the data and directly model the ratios of transcript quantification. The ANOVA allowed analysis with unequal variances for *CCL4*. Statistical significance was set at p = 0.05. Results from the qPCR analysis are summarized in Table 2 and Figure 2. In general, qPCR results followed the trend observed by microarray analysis, except for of *SLA-DQA*, which showed a pattern opposite to the one expected for the *0701/*04ns01 comparison. It appears that differences were more consistent between the *04ns01 and the two other groups, with *IL1B *transcript quantity reaching statistical significance (p = 0.05) and *TLR2 *(p = 0.0586) and *CASP1 *(p = 0.0651) approaching statistical significance, warranting further investigation. It is worth mentioning that lack of statistical significance does not automatically imply lack of biological significance. Analysis of variance components indicated that the individual pig (p = 0.05) was an important random effect in transcript quantification. This innate variability of individual pigs is most likely behind the lack of statistical significance in spite of fold-changes higher than 3 being observed. It also points out to the importance of including as many individuals as possible for qPCR confirmation of microarray data, especially individuals that have not been used for the microarray analysis, in order to obtain results that more closely reflect the situation in the population. In the case of *CCL4*, even though it appeared to be significantly differentially expressed in two of the comparisons (fold change > 3), the differences got lost within the high variability showed by the gene transcript quantifications in the tested groups, indicating that the differences may not be consistent at the population level. This seems to be also the case for differences observed between the *0502 and *0701 allele groups, a fact that suggests that the gene expression profiles of these two groups are not really that distinctive.

**Table 2:** Gene-specific primers and PCR conditions for relative quantification in the Light Cycler system

Gene name	GenBank^a^	Primers (5'-> 3') ^b^	Prod. size (bp) ^c^	Ann. temp. (°C) ^d^	Acq. temp. (°C) ^e^
*TLR2*	AB085935.1	F TGCGAATCCTGAAAATAGGC	343	59	84
		R CTTGCGTCAGTGATTTCTGC			
*CCL4*	NM_001075147	F GAAGCTCTGCGTGACTGTCC	391	59	87
		R AGGAACAGGATCTGCTGAGG			
*IL1B*	NM_214055.1	F GCAGATGGTGTCTGTCATCG	444	60	84
		R TTCTCCATGTCCCTCTTTGG			
*SLA-DQA*	AY191777.1	F TGTGGAGGTGAAGACATTGC	315	59	83
		R CAGCATCACTGGAGACTTGG			
*CASP1*	NM_214162.1	F GAGAAAATCTCACCGCTTCG	572	59	83
		R AGTCACTCTTTCGGCAGTGG			
*RPL19*	AV600389	F ATGAGACCAATGAAATCGCC	504	60	87
		R CATGAGGATCCGCTTGTTTT			

^a ^Sequenced used for primer design; ^b ^Sequence of forward (F) and reverse (R) primers in 5' to 3' orientation; ^c ^Size of the amplified PCR product; ^d ^Annealing temperature and ^e ^acquisition temperature for qPCR.

## List of abbreviations

*LTB*, Lymphotoxin beta (TNF superfamily, member 3); *BRD2*, Bromodomain containing 2; *CCL4*, Chemokine (C-C motif) ligand 4; *IL1B*, Interleukin 1 beta; *SLA-DQA*, SLA class II DQ alpha; *TLR2*, Toll-like receptor 2; *CASP1*, Caspase 1; *RPL19*, Ribosomal protein L19, EBI IPD, European Bioinformatics Institute Immuno-Polymorphism Database; BLAST, Basic Local Alignment Search Tool; RT-PCR, Reverse transcription – polymerase chain reaction; qPCR, quantitative PCR; SLA, Swine leukocyte antigen; MHC, Major histocompatibility complex; MNCs, Blood mononuclear cells; GEO, Gene Expression Omnibus; MIAME, Minimum information about a microarray experiment; LOWESS, Locally weighted scatter plot smoothing algorithm; FDR, False Discovery Rate.

## Competing interests

The authors declare that they have no competing interests.

## Authors' contributions

MINS participated in the conception and design of the study, carried out the qPCR confirmation, participated in the SLA-DRB1 allele characterization and drafted the manuscript. RJJ carried out the cDNA microarray hybridizations and helped draft the manuscript. BB established the SLA-defined lines of pigs and participated in the characterization of SLA-DRB1 alleles. BAM participated in the design and coordination of the project and helped to draft the manuscript. All authors read and approved the final manuscript.
